# Growth Hormone (GH) Treatment Decreases Plasma Kisspeptin Levels in GH-Deficient Adults with Prader–Willi Syndrome

**DOI:** 10.3390/jcm10143054

**Published:** 2021-07-09

**Authors:** Olga Giménez-Palop, Laia Casamitjana, Raquel Corripio, Susanna Esteba-Castillo, Rocío Pareja, Néstor Albiñana, Mercedes Rigla, Assumpta Caixàs

**Affiliations:** 1 Endocrinology and Nutrition Department, Hospital Universitari Parc Taulí, 08208 Sabadell, Spain; ogimenez@tauli.cat (O.G.-P.); lcasamitjana@tauli.cat (L.C.); rpareja@tauli.cat (R.P.); mrigla@tauli.cat (M.R.); 2 Institut d’Investigació i Innovació Parc Taulí (I3PT), 08208 Sabadell, Spain; rcorripio@tauli.cat (R.C.); nalbinana@tauli.cat (N.A.); 3 Department of Medicine, Universitat Autònoma de Barcelona, 08208 Sabadell, Spain; 4 Pediatrics Department, Hospital Universitari Parc Taulí, 08208 Sabadell, Spain; 5 Specialized Service in Mental Health and Intellectual Disability, Institut Assistència Sanitària (IAS), Parc Hospitalari Martí i Julià, 17190 Salt, Spain; susanna.esteba@ias.cat; 6 Neurodevelopment Group (Girona Biomedical Research Institute)—IDIBGI, Institute of Health Assistance (IAS), Parc Hospitalari Martí i Julià, 17190 Salt, Spain

**Keywords:** Prader–Willi Syndrome, kisspeptin, leptin, growth hormone deficiency

## Abstract

Obesity and growth hormone (GH)-deficiency are consistent features of Prader–Willi syndrome (PWS). Centrally, kisspeptin is involved in regulating reproductive function and can stimulate hypothalamic hormones such as GH. Peripherally, kisspeptin signaling influences energy and metabolic status. We evaluated the effect of 12-month GH treatment on plasma kisspeptin levels in 27 GH-deficient adult PWS patients and analyzed its relationship with metabolic and anthropometric changes. Twenty-seven matched obese subjects and 22 healthy subjects were also studied. Before treatment, plasma kisspeptin concentrations in PWS and obese subjects were similar (140.20 (23.5–156.8) pg/mL vs. 141.96 (113.9–165.6) pg/mL, respectively, *p =* 0.979)) and higher (*p =* 0.019) than in healthy subjects (124.58 (107.3–139.0) pg/mL); plasma leptin concentrations were similar in PWS and obese subjects (48.15 (28.80–67.10) ng/mL vs. 33.10 (20.50–67.30) ng/mL, respectively, *p =* 0.152) and higher (*p* < 0.001) than in healthy subjects (14.80 (11.37–67.30) ng/mL). After GH therapy, lean body mass increased 2.1% (*p =* 0.03), total fat mass decreased 1.6% (*p* = 0.005), and plasma kisspeptin decreased to levels observed in normal-weight subjects (125.1(106.2–153.4) pg/mL, *p =* 0.027). BMI and leptin levels remained unchanged. In conclusion, 12-month GH therapy improved body composition and decreased plasma kisspeptin in GH deficient adults with PWS. All data are expressed in median (interquartile range).

## 1. Introduction

Kisspeptin is a hormone that promotes the onset of puberty by stimulating the secretion of gonadotropin-stimulating hormone (GnRH). Kisspeptin can also stimulate the release of other pituitary hormones such as prolactin, growth hormone (GH), oxytocin, and vasopressin [1].

Kisspeptin is mainly synthesized by neurons in the arcuate nucleus of the hypothalamus. Its synthesis is modulated by energy balance, decreasing in situations of insufficient weight (e.g., anorexia nervosa) or excess weight (e.g., obesity) [2]. Low kisspeptin levels are considered the cause of the hypogonadotropic hypogonadism seen in some patients with metabolic syndrome and obesity [2,3].

Kisspeptin-producing neurons express receptors for leptin, a hormone produced in adipose tissue that informs these neurons about the status of energy reserves [4,5]. Kisspeptin is also expressed in peripheral tissues involved in metabolic functions such as the pancreas, liver, and adipose tissue, and emerging data suggests peripheral kisspeptin plays a role in the regulation of insulin secretion [6].

Prader–Willi Syndrome (PWS) is a rare genetic disease characterized by a series of disorders of hypothalamic functionalism: hyperphagia, obesity, hypogonadotropic hypogonadism, short stature due to GH deficiency [7,8,9], and cognitive, praxis, and behavioral disorders [10,11,12]. Up to 60% of adults with PWS have GH deficiency [13], which results in decreased muscle mass and strength and increased fat mass [14]. These changes can be mitigated with GH treatment, which induces metabolically beneficial changes in body composition [15,16].

Kisspeptin concentrations are decreased in patients with idiopathic hypogonadotropic hypogonadism, diabetes, or metabolic syndrome [2,4,5]. To our knowledge, kisspeptin has not been studied in patients with PWS; however, since these patients have impaired hypothalamic function, obesity (often associated with diabetes), central hypogonadism, and GH deficiency, they would be expected to have decreased kisspeptin levels. Moreover, it remains to be determined whether GH treatment could influence kisspeptin synthesis directly through a negative feedback mechanism or indirectly through changes in body composition.

We aimed to determine changes in plasma kisspeptin concentrations in 27 adult patients with PWS after 12 months of GH treatment and whether these changes are associated with changes in anthropometric or metabolic parameters. 

## 2. Materials and Methods

### 2.1. Subjects

We included 27 adults with PWS (15 women, median age: 26 years, range: 18–53) treated at our center between 1 January 2016 and 31 January 2019. Cytogenetic analysis revealed 7 had type 1 deletion, 10 had type 2 deletion, 6 had maternal uniparental disomy, 3 had imprinting defects and 1 had an atypical BP2-BP4 microdeletion. All had GH deficiency diagnosed with GHRH-arginine and/or glucagon stimulation tests [17,18,19]. The cut off for GH deficiency with GHRH-arginine test was: GH < 11 ng/mL if BMI < 25 kg/m^2^, <8 ng/mL if BMI 25–30 kg/m^2^, and <4 ng/mL if BMI ≥ 30 kg/m^2^. The cut off for glucagon test was GH < 3 ng/mL at any time point.

Six patients had type 2 diabetes with good glycemic control (HbA1c < 7.5%) and 15 (7 women) were receiving sex steroids. None had precocious puberty. For comparison, we also evaluated 27 obese subjects matched for age, sex, and BMI and 22 healthy subjects.

The study complied with all provisions in the Declaration of Helsinki and was approved by the local ethics committee (Comitè d’Ètica d’Investigació amb medicaments del Parc Taulí). All patients with PWS agreed to participate after being informed together with their parents or caregivers; their legal guardians (usually their parents) signed the consent form before enrollment. All control participants provided written informed consent.

### 2.2. Methods

Blood was extracted from all participants at 8 AM after overnight fasting. Plasma samples for kisspeptin and leptin measurements were kept at −80 °C until analysis. We recorded subjects’ height, determined by a stadiometer (Harpenden, Holtain Ltd., Dyfed, UK); body weight, measured to the nearest 0.1 kg with standard equipment; body mass index (BMI); and body composition determined by dual-energy x-ray absorptiometry (Lunar Prodigy−963, Chicago, IL, USA).

To determine concentrations of kisspeptin-1 in plasma, we used the Kisspeptin-1 ELISA kit (Cloud-Clone Corp., Houston, TX, USA) (lower limit of detection, 9.27 pg/mL; intraassay coefficient of variation (CV) <10%; interassay CV < 12%). To determine concentrations of leptin in plasma, we used the Human Leptin ELISA Kit (Biorbyt, Cambridge, UK) (lower limit of detection, 10 pg/mL; intraassay CV < 7.6%; interassay CV < 8.4%). A routine automated analyzer was used for other laboratory tests. The homeostatic model assessment for insulin resistance (HOMA-IR) index was calculated as fasting plasma glucose (mmol/L) × fasting insulin (µIU/mL)/22.5 [20]. 

Patients with PWS were treated with recombinant GH (Genotonorm Miniquick^®^, Pfizer, New York, NY, USA), starting with a dose of 0.2 mg/day and adjusting the dose at 1, 3, 6, and 12 months to achieve high-normal insulin-like growth factor-1 (IGF-1) levels for the patient’s age.

Only PWS patients received GH treatment. After 12 months’ GH treatment, patients’ analytic and anthropometric parameters were measured again with the same protocol.

### 2.3. Statistical Analyses 

Continuous variables are reported as medians and interquartile ranges (IQR). Categorical variables are reported as frequencies and percentages. To compare all the continuous variables at baseline, between the 3 groups, we used Kruskal–Wallis test followed by the Mann–Whitney U test with Bonferroni correction. To compare all variables before and after GH treatment we used the Wilcoxon signed-rank test. To study the relationship between variables, we used Spearman’s rank-order correlation test. Statistical significance was fixed at *p* < 0.05. All analyses were done with IBM SPSS Statistics for Windows, version 25.0 (IBM Corp., Armonk, NY, USA).

## 3. Results

### 3.1. Baseline Findings

#### 3.1.1. Patient Characteristics

Table 1 reports the baseline characteristics of subjects in the PWS, obese, and normal weight groups. The PWS and obese groups did not differ in weight, BMI, waist, percentages of body fat and lean mass, glucose, HOMA-IR, follicle-stimulating hormone, or testosterone (only measured in men) levels. Females in the PWS group had lower levels of estradiol and luteinizing hormone than females in the obese group. 

#### 3.1.2. Kisspeptin Levels

Overall, kisspeptin levels did not differ between sexes (132.9 pg/mL (107.8–161.8) in females vs. 135.3 pg/mL (119.9–162.2) in males, *p* = 0.488).

**Figure 1:**
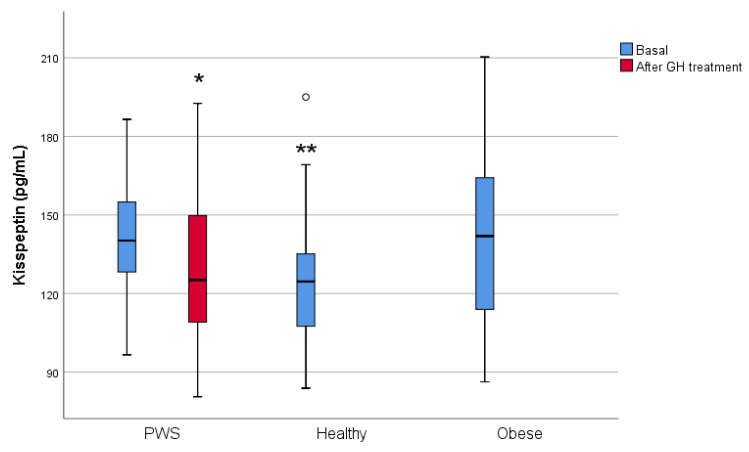
Kisspeptin levels at baseline in the three groups (blue) and after GH treatment in PWS (red). GH = growth hormone. * Willcoxon signed-rank test, *p =* 0.027 between baseline and post-GH treatment. ** Mann–Whitney U test with Bonferroni correction, *p =* 0.019 between PWS and healthy groups. Outliers are represented as circle symbols.

In PWS subjects, baseline kisspeptin levels were similar to those in the obese group and higher than those in the healthy group (Table 1, Figure 1). There were no differences between diabetic and non-diabetic subjects (141.1 pg/mL (121.1–152.6) vs. 140.2 pg/mL (128.2–163.6), respectively, *p* = 0.798). Patients treated with sex steroids had higher levels of kisspeptin (149.4 pg/mL (138.3–165.3) vs. 133.6 pg/mL (117.8–142.2) than those not treated with sex steroids, *p* = 0.01), but these two groups did not differ in terms of BMI (*p* = 0.981), testosterone levels in men, or estradiol levels in women (data not shown).

#### 3.1.3. Leptin Levels

Overall, leptin levels were higher in females than in males (43.95 ng/mL (22.75–73.05) vs. 22.80 ng/mL (11.80–29.50) respectively, *p* < 0.001).

**Figure 2:**
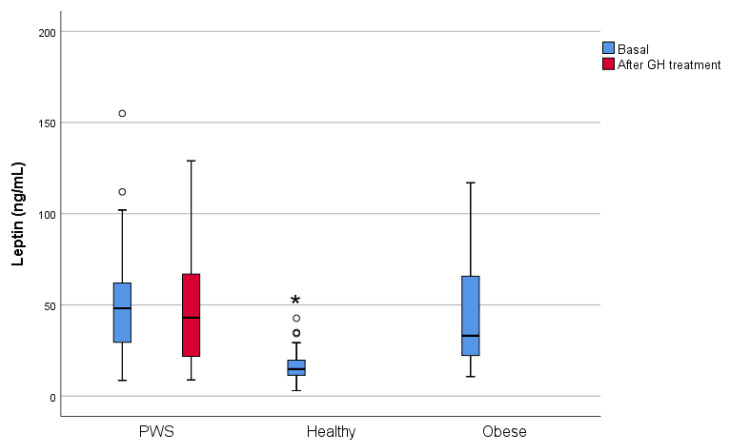
Leptin levels at baseline in the three groups (blue) and after GH treatment in PWS (red).* *p* < 0.001 Differences in baseline leptin levels between the three groups (Kruskall–Wallis test). Leptin levels did not change in Prader–Willi subjects after GH treatment (Willcoxon signed-rank test, *p* = 0.144). Outliers are represented as circle symbols.

In PWS subjects, baseline leptin levels were similar to those in the obese group and higher than those in the healthy group (Table 1, Figure 2). Leptin levels did not differ between diabetic and non-diabetic subjects (45.30 ng/mL (19.60–61.80) vs. 51.0 ng/mL (29.50–82.40), respectively, *p* = 0.866) or between those treated with sex steroids and those not treated with sex steroids (51.0 ng/mL (31.20–62.0) vs. 45.2.0 ng/mL (26.70–82.40), respectively, *p* = 0.919).

### 3.2. Comparison between Baseline and Post-GH-Treatment Findings in the PWS Group

After GH treatment, no significant changes were observed in anthropometric measures, glucose metabolism, gonadotropins, or sex steroids. As expected, IGF-1 levels increased (143(95–188) ng/mL vs. 217(160–254) ng/mL, *p* < 0.001) (Table 2). Kisspeptin levels were significantly lower than at baseline (*p* = 0.027) and were similar to those in the healthy group (125.1 pg/mL (106.2–153.4) vs. 124.58 pg/mL (107.3–139.0), respectively, *p* = 0.936) (Table 2, Figure 1). Body composition improved after treatment: total body fat was 1.6% lower (*p =* 0.005) and lean body mass was 2.1% higher (*p =* 0.005) (Table 2).

By contrast, leptin levels were not significantly different after treatment (Table 2, Figure 2) and no significant changes in bone mineral density were observed; Z-scores remained lower than expected for age (Table 2).

## 4. Correlations 

Overall, baseline kisspeptin did not correlate significantly with weight, BMI, percentage of body fat or total body fat, percentage of lean mass or total lean mass, waist circumference, glucose, insulin, HOMA-IR, glycated hemoglobin, gonadotropins, or sex steroids. Baseline kisspeptin and leptin levels correlated only in the healthy group (r = 0.414, *p =* 0.05).

In the PWS group, baseline kisspeptin did not correlate significantly with any of the above variables or with IGF-1, bone mineral density (total femur Z-score, total spine Z-score), extremities/trunk body fat index, or appendicular skeletal muscle mass index. Moreover, the percentage of change in kisspeptin did not correlate with the percentage of change in body fat (whether measured in % or kg), lean body mass (whether measured in % or kg), HOMA-IR, insulin, glucose, or IGF-1. 

Overall, baseline leptin levels correlated with percentage of body fat (r = 0.721, *p* < 0.001) and total body fat (r = 0.762, *p* < 0.001).

In the PWS group, baseline leptin levels correlated with HOMA-IR (r = 0.317, *p* = 0.006), but after GH treatment, leptin levels did not correlate with HOMA-IR (r = 0.110, *p* = 0.602). Baseline leptin levels also correlated with percentage of body fat (r = 0.705, *p* < 0.001) and after treatment, with percentage of body fat (r = 0.692, *p* < 0.001), as well as with total body fat (r = 0.498, *p* = 0.01).

The percentage of change in IGF-1 did not correlate with the percentage of change in total body fat or in lean body mass.

## 5. Discussion

To our knowledge, this is the first study to evaluate plasma kisspeptin levels in PWS patients. We found that plasma kisspeptin levels in PWS patients with GH deficiency were similar to those in obese subjects matched by age, sex, and BMI and were higher than those in healthy controls. After 12 months of treatment with GH, plasma kisspeptin levels in PWS patients decreased to levels similar to those observed in healthy subjects. 

The relationship between obesity and kisspeptin levels is unclear. Hestiantoro et al. [21] found kisspeptin levels were lower in obese than in normal-weight menopausal women. Pita et al. [22] found kisspeptin levels were higher in obese than in normal-weight prepubertal girls, but similar in obese and normal-weight prepubertal boys. Sitticharoon et al. [23] observed higher kisspeptin levels in obese than in non-obese men. In other studies, this group found no differences in kisspeptin levels between obese and normal-weight women [24] or between obese and normal-weight girls with central precocious puberty [25]. Taken together, these findings suggest that kisspeptin levels may be influenced more by hormonal status than by obesity per se. Moreover, kisspeptin levels in women differ across the menstrual cycle, being highest in the luteal phase, followed by the preovulation phase and the follicular phase, suggesting that kisspeptin might play a role in peripheral reproductive regulation [26]. In the present study, kisspeptin levels were higher in obese patients (with or without PWS), but did not differ between sexes. However, all our PWS patients had some degree of hypogonadism, and not all of them were being treated with sex hormones. Furthermore, we did not take women’s menstrual phase into account because most of these patients do not ovulate. Nevertheless, kisspeptin levels were higher in those with hormone replacement therapy than in those without, suggesting sex hormones might affect peripheral kisspeptin. These findings are in line with those reported in vitro experiments where estrogens stimulated *Kiss-1* gene expression in cultured kisspeptin neurons, suggesting a central interaction between sex hormones and kisspeptin-producing cells [27].

The relationship between kisspeptin and BMI is also unclear. Some studies have found positive correlations. One study in men found that kisspeptin levels correlated positively with BMI and weight [23], and another in children found they correlated positively with BMI, weight, and waist circumference [28]. However, other studies have found negative correlations between kisspeptin levels and BMI and waist circumference in non-diabetic men and women [29] and between kisspeptin and BMI in anorectic women [30]. In the present study, we found no correlations between kisspeptin and BMI or waist circumference in the whole group or in the PWS group. Discrepancies between studies might be due to the inaccuracy of BMI and waist circumference as measures of body fat and/or to the heterogeneity and the small size of the cohorts studied.

In the PWS group, GH treatment resulted in a decrease in total body fat and an increase in lean body mass, as reported by other authors [31,32]. Because adipocytes appear to be a source of circulating kisspeptin, we expected decreases in body fat mass to result in decreases in kisspeptin levels. Moreover, adipocytes express the kisspeptin-1 receptor (KISS1R), indicating that kisspeptin secreted by adipose tissue could act as an adipokine or as autocrine/paracrine regulator of adipocyte function [33]. However, we found no correlations between the change in kisspeptin and the change in fat mass, lean mass, extremities/trunk body fat index, or appendicular lean mass index after treatment, suggesting there is no clear relationship between kisspeptin and body composition parameters. Furthermore, kisspeptin appears to regulate GH, but the role of kisspeptin in GH release remains unclear, especially due to discrepancies between in vivo and in vitro findings [1], although the data suggest that *kiss1/KISS1R* could play a role in a short or ultra-short feedback loop that regulates the function of somatotrophs [1]. Such a role would help explain why kisspeptin levels decreased in GH-deficient patients with PWS after one year of GH therapy. Nevertheless, we found no correlation between the changes in kisspeptin and IGF-1 levels. Further studies are necessary to test this hypothesis. 

Leptin is mainly produced by adipose tissue [34], and its concentration in serum correlates with body energy reserves [35]. Leptin may signal peripheral energy status to the hypothalamus, thus influencing food intake and reproductive function [36]. Kisspeptin neurons in the hypothalamus have leptin receptors, and leptin increases *Kiss1* mRNA expression in those neurons, thus supporting the existence of a leptin-kisspeptin-GnRH pathway in which kisspeptin would mediate between energy reserves and the maturation of the hypothalamic-pituitary-gonadal axis [2].

In our study, plasma leptin levels correlated with body fat mass in all groups at baseline, and no significant changes in leptin levels occurred in the PWS group after GH treatment. These findings are in line with those reported by Höybe et al. [37] in 2003 in a study with 17 adult patients with PWS, but discrepant with those reported by Myers et al. [38], who reported decreased leptin levels after GH treatment in children with PWS. Moreover, we found no correlation between plasma leptin and kisspeptin levels, indicating that these two peptides could be less interrelated peripherally than in the central nervous system.

Inactivation of kiss1r in mice results in obesity and diabetic phenotype [39], and kisspeptin and its receptor are expressed in metabolic tissues (e.g., fat, liver and pancreatic tissues) and likely plays a role in regulating insulin secretion. [6]. However, kisspeptin stimulates insulin secretion only when glucose levels are elevated, suggesting kisspeptin’s role involves correcting hyperglycemia [40]. Glucagon secreted from pancreatic α-cells provokes kiss1 expression in the liver, increasing secretion of kisspeptin from the liver and thereby suppressing glucose-stimulated insulin secretion from pancreatic β-cells [33].

For all these reasons, we also analyzed the relationship between kisspeptin and glucose metabolism in our cohort of patients with PWS, 6 (22.2%) of whom had diabetes with good glycemic control (HbA1c < 7.5%). We found no difference in kisspeptin levels between diabetic and non-diabetic PWS patients, and kisspeptin levels did not correlate with plasma glucose, insulin, glycated hemoglobin, or HOMA-IR index. After GH treatment, no changes in these parameters or correlations between these parameters and kisspeptin were observed. These results corroborate those of a previous study that showed no effect of GH treatment on glycemic control in adults with PWS [32]. 

The limitations of our study are mainly due to the characteristics of the sample. The small size of the groups precluded subanalyses by sex or by genetic subtype. Moreover, in both the PWS group and the matched obese controls, obesity was heterogeneous, ranging from low risk (class 1) to high risk (class 3). Only a small proportion of patients were receiving sex hormones, and we did not take the menstrual cycle phase into consideration. All these limitations may have contributed to the lack of significant correlations between variables, and larger studies would be necessary to draw conclusions.

The authors want to emphasize that the recent development of more sensitive methods to detect plasma kisspeptin concentrations leading to better quantitative results instead of undetectable values may have influenced the opposite findings to the initial hypothesis of the present study.

In summary, we found that plasma kisspeptin levels in our cohort of GH-deficient adults with PWS did not differ from those in matched obese patients and were higher than those in a group of normal weight subjects. After one year of GH treatment, kisspeptin levels decreased to levels similar to those in healthy controls. Although the mechanism through which this decrease occurs and its clinical significance remain to be determined, we speculate that GH treatment might decrease kisspeptin levels directly through a negative feedback mechanism, since we found no relationship between changes in kisspeptin levels and changes in anthropometric or metabolic parameters or in leptin levels. Further studies are required to corroborate this hypothesis.

## Figures and Tables

**Table 1:** Baseline characteristics of participants.

	PWS Patients (*n* = 27)	Obese Subjects (*n* = 27)	Healthy Subjects (*n* = 22)	*p*-Value
Sex (female)	15 (55.6%)	15 (55.6%)	11 (50%)	* 0.907
Age (years)	26 (24–37)	28 (24–37)	27.5 (21.5–37.5)	* 0.909
Weight (kg)	89.6 (70.5–105.5)	95.5 (81.8–121.1)	65.8 (58.1–71.6)	* <0.001 ⴕ 0.135 λ <0.001 ** <0.001
Height (cm)	156 (148.0–165.0)	169 (162.0–173.0)	169 (164.5–181.5)	* <0.001 ⴕ <0.001 λ <0.001 ** 0.344
BMI (kg/m^2^)	34.6 (30.9–41.3)	32.1 (29.3–41.9)	22.2 (20.9–22.9)	* <0.001 ⴕ 0.580 λ <0.001 ** <0.001
Waist (cm)	110.0 (101.0–124.0)	107.0 (99.0–121.0)	78.5 (71.5–83.7)	* <0.001 ⴕ 0.387 λ <0.001 ** <0.001
Glucose (mmol/L)	5.00 (4.55–6.99)	4.83 (4.77–5.49)	4.36 (4.16–5.02)	* 0.006 ⴕ 0.827 λ 0.004 ** 0.006
HOMA-IR	2.26 (1.52–4.80)	2.58 (1.79–3.96)	1.37 (0.84–1.84)	* <0.001 ⴕ 0.710 λ 0.001 ** <0.001
IGF-I (ng/mL)	143.0 (95.0–188.0)	171 (139.5–257.5)	226.0 (192.7–312.0)	* <0.001 ⴕ =0.007 λ <0.001 ** 0.084
LH (IU/L)	1.21 (0,36–6.29)	6.01 (4.1–7.75)	5.48 (3.5–12.75)	* 0.001 ⴕ 0.001 λ 0.001 ** 0.947
FSH (IU/L)	3.63 (0.3–7.28)	5.19 (2.9–6.76)	3.31 (2.3–7.22)	* 0.301
Testosterone, in males (ng/mL)	2.08 (0.51–3.61)	3.82 (2.47–4.44)	5.63(4.82–8.11)	* <0.001 ⴕ 0.75 λ <0.001 ** 0.002
Estradiol, in females (pg/mL)	23 (16–44)	94 (60–164)	63 (5–168)	* 0.001 ⴕ <0.001 λ 0.253 ** 0.287
Fat mass (%)	56.3 (49.3–61.1)	50.8 (41.9–61.9)	38.7(28.1–39.5)	* <0.001 ⴕ 0.640 λ <0.001 ** <0.001
Lean mass (%)	43.6(38.9–50.2)	49.2 (36.4–51.3)	69.2 (56.4–71.8)	* < 0.001 ⴕ 0.891 λ <0.001 ** <0.001
Kisspeptin (pg/mL)	140.20 (123.5–156.8)	141.96 (113.9–165.6)	124.58 (107.3–139.0)	* 0.094 ⴕ 0.979 λ 0.019 ** 0.154
Leptin (ng/mL)	48.15 (28.80–67.10)	33.10 (20.50–67.30)	14.8 (11.4–67.3)	* <0.001 ⴕ 0.152 λ <0.001 ** <0.001

PWS = Prader–Willi syndrome; BMI = body mass index; HOMAR-IR = homeostatic model assessment for insulin resistance; LH = luteinizing hormone; FSH = follicle-stimulating hormone. *p*-values are marked with * Kruskall–Wallis test for comparisons of PWS vs. Obese vs. Healthy. Mann–Whitney U test with Bonferroni correction for the following comparisons: ⴕ PWS vs. Obese, λ PWS vs. Healthy, and ** Obese vs. Healthy. All variables except for sex are reported as median (interquartile range).

**Table 2:** Anthropometrics, body composition, and plasma hormone levels in patients with PWS before and after 12 months’ growth hormone treatment.

	Before GH Treatment Median (IQR)	After 12 Months’ GH Treatment Median (IQR)	*p*-Value *
Weight (kg)	89.6 (70.5–105.5)	87.7 (74.3–100.2)	0.990
BMI (kg/m^2^)	34.6 (30.9–41.3)	34.0 (31.8–41.6)	0.692
Waist (cm)	110.0 (101.0–124.0)	112.0 (104.0–123.5)	0.602
Glucose (mmol/L)	5.00 (4.55–6.99)	4.83 (4.33–5.49)	0.107
HOMA-IR	2.26 (1.52–4.80)	2.95 (1.99–6.14)	0.209
HbA1c (%)	5.6 (5.3–6.9)	5.6 (5.3–6.2)	0.294
LH (IU/L)	1.21 (0.36–6.29)	2.71(0.3–5.81)	0.841
FSH (IU/L)	3.63 (0.3–7.28)	5.82 (0.92–9.03)	0.015
Testosterone, in males (ng/mL)	2.08 (0.51–3.61)	1.43 (0.43–3.22)	0.814
Estradiol, in females (pg/mL)	23 (16–44)	16 (8–31)	0.124
IGF-1 (ng/mL)	143(95–188)	217 (160–254)	<0.001
Fat mass (%)	56.3 (49.3–61.1)	52.1 (49.9–59.2)	0.028
Lean mass (%)	43.6(38.9–50.2)	47.9 (40.8–50.1)	0.002
Total body water (kg)	37.6(32.2–43.0)	37.1 (32.9–43.6)	0.342
Extremities/trunk body fat index	0.92(0.70–1.09)	0.86 (0.75–1.11)	0.637
Appendicular skeletal muscle mass index (kg/m^2^)	6.6(5.6–7.7)	7.2 (6.1–8.4)	0.059
Total femur bone mineral density (Z-score)	−1.31(−1.8–(−0.65))	−1.22 (−1.77–(−0.43))	0.485
Total spine bone mineral density Z-score	−1.61(−2.20–(−0.56))	−1.90 (−2.49–(−0.51))	0.927
Kisspeptin (pg/mL)	140.20 (123.5–156.8)	125.1 (106.2–153.4)	0.027
Leptin (ng/mL)	48.15 (28.80–67.10)	43.00 (21.75–68.32)	0.144

IQR = interquartile range; BMI = body mass index; HOMAR-IR = homeostatic model assessment for insulin resistance; LH = luteinizing hormone; FSH = follicle-stimulating hormone; IGF−1 = insulin-like growth factor−1. * Willcoxon signed-rank test.

## Data Availability

Data supporting reported results can be found in Parc Taulí server (contact jcoliva@tauli.cat or the corresponding author acaixas@tauli.cat).
